# Compartment-specific ^129^Xe HyperCEST z spectroscopy and chemical shift imaging of cucurbit[6]uril in spontaneously breathing rats^☆^

**DOI:** 10.1016/j.zemedi.2023.08.005

**Published:** 2023-09-01

**Authors:** Agilo Luitger Kern, Marcel Gutberlet, Regina Rumpel, Inga Bruesch, Jens M. Hohlfeld, Frank Wacker, Bennet Hensen

**Affiliations:** a Institute for Diagnostic and Interventional Radiology, Hannover Medical School, Carl-Neuberg-Straße 1, 30625 Hannover, Germany; b Biomedical Research in Endstage and Obstructive Lung Disease Hannover (BREATH), German Center for Lung Research (DZL), Carl-Neuberg-Straße 1, 30625 Hannover, Germany; c Institute for Laboratory Animal Science and Central Animal Facility, Hannover Medical School, Carl-Neuberg-Straße 1, 30625 Hannover, Germany; d Clinical Airway Research, Fraunhofer Institute for Toxicology and Experimental Medicine (ITEM), Nikolai-Fuchs-Straße 1, 30625 Hannover, Germany; e Department of Respiratory Medicine, Hannover Medical School, Carl-Neuberg-Straße 1, 30625 Hannover, Germany

**Keywords:** ^129^Xe MRI, HyperCEST, z spectroscopy, Chemical shift imaging, Cucurbit[6]uril, Small animal

## Abstract

^129^Xe hyperpolarized gas chemical exchange saturation transfer (HyperCEST) MRI has been suggested as molecular imaging modality but translation to in vivo imaging has been slow, likely due to difficulties of synthesizing suitable molecules. Cucurbit[6]uril–either in readily available non-functionalized or potentially in functionalized form–may, combined with ^129^Xe HyperCEST MRI, prove useful as a switchable ^129^Xe MR contrast agent but the likely differential properties of contrast generation in individual chemical compartments as well as the influence of ^129^Xe signal drifts encountered in vivo on HyperCEST MRI are unknown. Here, HyperCEST z spectroscopy and chemical shift imaging with compartment-specific analysis are performed in a total of 10 rats using cucurbit[6]uril injected i.v. and under a protocol employing spontaneous respiration. Differences in intensity of the HyperCEST effect between chemical compartments and anatomical regions are investigated. Strategies to mitigate influence of signal instabilities associated with drifts in physiological parameters are developed. It is shown that presence of cucurbit[6]uril can be readily detected under spontaneous ^129^Xe inhalation mostly in aqueous tissues further away from the lung. Differences of effect intensity in individual regions and compartments must be considered in HyperCEST data interpretation. In particular, there seems to be almost no effect in lipids. ^129^Xe HyperCEST MR measurements utilizing spontaneous respiration protocols and extended measurement times are feasible. HyperCEST MRI of non-functionalized cucurbit[6]uril may create contrast between anatomical structures in vivo.

## 1 Introduction

Although the introduction of hyperpolarized ^129^Xe MRI to the field of biomedical imaging was historically motivated by imaging applications outside the lung, in particular by imaging the brain [1], [2], most of the later work has focused on the study of lung function as well as lung microstructure [3], [4], [5], [6], [7], [8], [9]. Clinical translation of ^129^Xe MRI for routine assessment of lung ventilation in patients with lung diseases including clearance of this technology by the U.S. Food and Drug Administration (FDA) [10] has been achieved in recent years although still at a small scale in selected centers [11].

The improvement of ^129^Xe hyperpolarization technology with respect to polarization level and gas throughput over the last years may have lead to renewed interest in the development of imaging applications for organs outside the lung. MRI of brain and kidneys is feasible in humans at acceptable image resolution already after inhalation of single gas doses with subsequent breathhold [12], [13] despite the fairly short $T_{1}$ of ^129^Xe in solution at clinical fields [14], [15]. Also multiple inhalations of ^129^Xe are feasible and maybe even more tolerable in humans [16]. Further improvements in hyperpolarization methods are necessary, however, for supplying ^129^Xe during extended imaging sessions as they are common in conventional ^1^H MRI. The in principle probably optimal setup employing real-time ^129^Xe production is even more difficult in practice and associated with SNR penalties [17], [18]. Consequently, so far, MR imaging of small animals remains the natural testbed for novel methods requiring acquisition times longer than several breathholds.

Molecular imaging in hyperpolarized gas MRI of the lung has been achieved by application of paramagnetic nanoparticles [19], [20], [21]. In addition, ^129^Xe HyperCEST has been proposed as tool for ultrasensitive detection of dissolved and potentially functionalized molecules within living systems using MRI [22]. Here, saturating chemically shifted ^129^Xe spins transiently encapsulated in cage-like molecules leads to a reduction of the much larger magnetization in a solvent compartment, leading to improved detection sensitivity compared to direct excitation of bound ^129^Xe. Among the potential advantages of this method over established molecular imaging methods are the lack of ionizing radiation as well as the fact that image resolution is in principle merely limited by acquisition time in MRI. The translation of initially promising in vitro results to in vivo imaging has been unexpectedly slow, however, for various reasons, among them being the difficulty to synthesize suitable, non-toxic and functionalized molecules.

Cucurbit[n]urils are thought to be non-toxic macrocyclic molecules with potential for various medical applications. In particular, the cucurbit[n]urils with n = 7 and 8 have been found to be non-toxic at concentrations up to 1 mM. [23] In addition, the non-functionalized cucurbit[n]uril family member cucurbit[6]uril (CB6) has been shown to be detectable using HyperCEST in rats [24], [25] and thus may prove useful for in vivo methods development. Apart from classical molecular imaging applications, it may potentially also serve as a contrast agent in ^129^Xe MRI similar to established gadolinium-based contrast agents modulating longitudinal magnetization in ^1^H MRI. This however with the added advantage of being switchable through the HyperCEST effect.

HyperCEST differs in its properties from conventional CEST imaging in ^1^H MRI in that $T_{1}$ relaxation always decreases the magnetization to an equilibrium value of essentially zero. This effect limits the useful lengths of RF saturation pulse trains for contrast generation [26]. On the other hand, the relative signal difference should always increase with saturation time. In addition, since polarization levels obtained at a specific imaging location depend not only on the experimental setup but also on physiological parameters, large baseline signal drifts may complicate the interpretation of HyperCEST imaging results in vivo.

Previous work used controlled ventilation of the animal for providing a ^129^Xe magnetization as constant as possible to enable HyperCEST detection of CB6 [25]. Such an approach would be problematic, however, in terms of the ultimate goal of clinical translation, where free-breathing protocols with accompanying signal drifts would likely be used.

The fact that in vivo ^129^Xe dissolved in different compartments like lipids, aqueous tissues or red blood cells is associated with several distinct resonances [27] with probably variable exchange kinetics and also exchange amongst each other versus a single resonance in simple in vitro experiments is likely to complicate the situation. However, little is known about the effects of such complications on the HyperCEST contrast in general and the one created by CB6 within the different chemical compartments encountered in vivo in particular.

Thus, purpose of this work was to assess the feasibility of ^129^Xe HyperCEST MRI using cucurbit[6]uril as contrast agent under spontaneous respiration in rats and to investigate possible differences in individual chemical compartments using z spectroscopy and chemical shift imaging (CSI).

## 2 Materials and methods

### 2.1 Animal preparation

This study was conducted according to the German animal welfare act (TierSchG) and the European Directive 2010/63/EU and was approved by the Lower Saxony State Office for Consumer Protection and Food Safety (LAVES, AZ-20/3501). 10 female LEWIS rats (209 g ± 13 g, Charles River, Wilmington, USA) were used.

Similarly as in [28], animals were initially placed in a closed chamber and anesthetized using 4% isoflurane in oxygen flowing at 3 L/min. After induction of anesthesia, animals were positioned on an animal cradle within the RF coil and isoflurane flow directed through a nose cone (∼2% at 1 L/min). A respiration pillow and a rectal temperature sensor were placed for physiological monitoring and triggering of the MRI (Model 1030, Small Animal Instruments, Inc., Stony Brook, USA). Warm water was circulated through a heating blanket positioned beneath the animal to maintain body temperature (Single Pump Fluid Heating System, Small Animal Instruments, Inc.). A tail vein catheter was placed.

CB6 (Sigma–Aldrich, St. Louis, USA) was dissolved in phosphate-buffered saline at concentration 5 mM. The solution was heated and stirred and pushed through a 0.45 μm syringe filter. The solution was of clear appearance after filtration. Between HyperCEST z spectroscopy experiments, 10 mL/kg body weight of the solution were slowly injected into the tail vein of the animal.

### 2.2 ^129^Xe administration

**Figure 1:**
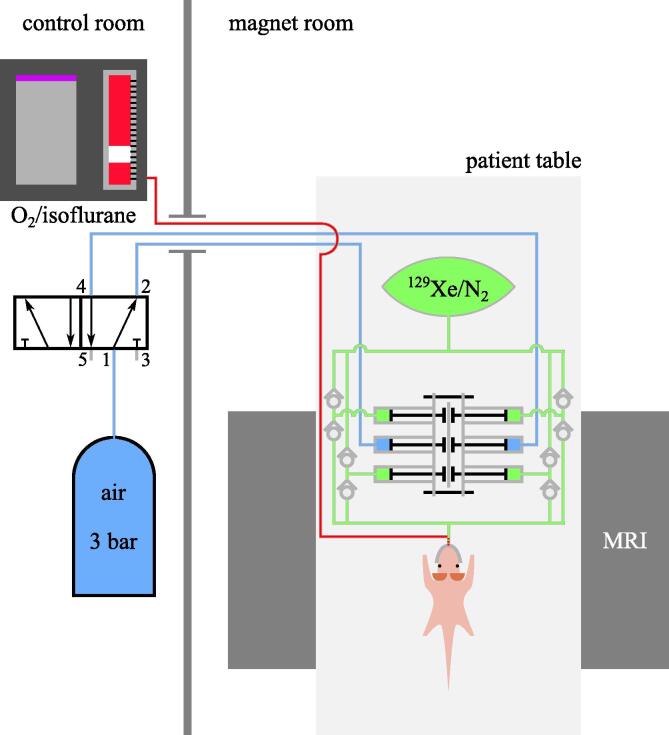
Schematic of the experimental setup with gas delivery device. Components not MR compatible are located within the control room and pneumatic/oxygen tubing is routed through a waveguide.

For each of the three measurements (z spectroscopy before CB6, after CB6 and HyperCEST CSI) a fresh batch of 500 mL of ^129^Xe were hyperpolarized to 20–30% (Polarean 9810, Polarean, Durham, USA), dispensed in 3 L/1 L Tedlar bags for z spectroscopy/CSI, respectively, and balanced with N_2_ in order to allow for extended measurement times at given flow and reduce longitudinal relaxation. Gas bags were attached to a home-made device for administration through the nose cone at an average flow of ∼100 mL/min. The device features plastic syringes connected by threaded rods to form a set of connected double-acting cylinders. Both sides of the inner cylinder are alternatingly pressurized with 3 bar air pressure and vented to atmosphere using a solenoid 5/2-way valve controlled by a programmable timing relay. Plastic check valves restrict direction of gas flow created by outer cylinders. Compared to a previous design [28], this design does not restrict gas volume and offers improved long-term stability, which is important for the HyperCEST application. It comes at the expense of an oscillatory flow component, however, with periodic time (here: 750 ms) short compared to the TRs used. A sketch of the experimental setup is shown in Fig. 1. During ^129^Xe imaging experiments, flow of oxygen was reduced to ∼400 mL/min.

### 2.3 MR imaging and spectroscopy

Imaging was performed at 2.89 T (Magnetom Vida, Siemens Healthineers, Erlangen, Germany) using the built-in gradient coils and a dedicated rat birdcage RF coil dual-tuned to the ^1^H and ^129^Xe resonances at 123.2 MHz and 34.1 MHz, respectively (Rapid Biomedical, Rimpar, Germany). Rats were placed with kidneys in the RF coil center. Transmitter calibration assumed a constant reference amplitude for all animals based on values determined within the lung in previous experiments. Frequency calibration for ^129^Xe MRI was performed based on ^1^H signals assuming a fixed ratio of Larmor frequencies [27].

#### 2.3.1 HyperCEST z spectroscopy

**Figure 2:**
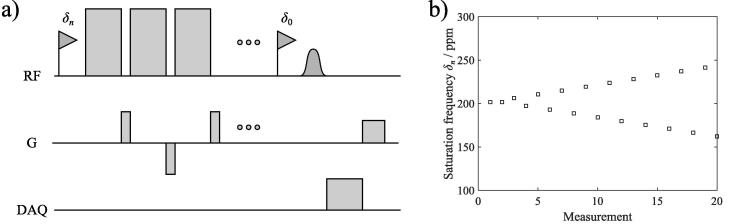
a) Sequence diagram of HyperCEST z spectroscopy sequence (dimensions not to scale). b) The saturation frequency $\delta_{n}$ is altered from the frequency of the dissolved phase in a centric fashion in z spectroscopy.

A HyperCEST z spectroscopy was implemented according to the sequence diagram shown in Fig. 2. z spectroscopy was performed in all rats before and after intravenous injection of CB6 solution. 20 rectangular 50 ms long saturation pulses (∼24 μT) separated by 1 ms long gaps were used. Saturation frequency was alternatingly shifted upfield and downfield from the dissolved phase (∼202 ppm), increasing absolute shift in 4.4 ppm steps, maximum shift 220 ppm. Magnetization was excited with a 500 μs long Gaussian pulse. Other sequence parameters included: $T_{R}$ 10 s, flip angle (excitation) 90°, receiver bandwidth 30 kHz, 1024 spectral points, 2 averages (long-term).

#### 2.3.2 HyperCEST CSI

**Figure 3:**
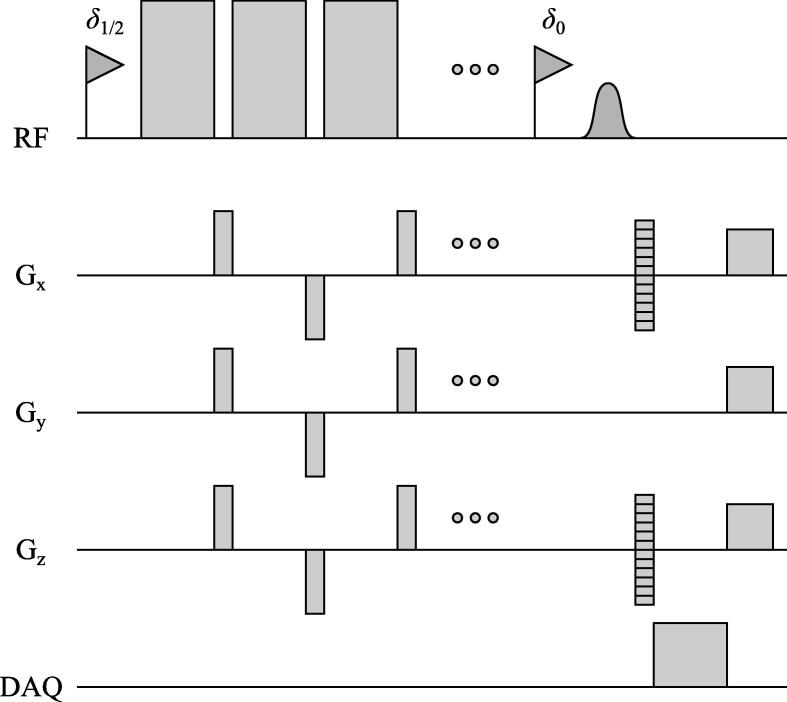
a) Sequence diagram of HyperCEST CSI sequence (dimensions not to scale). The saturation frequency is alternated between $\delta_{1}$ and $\delta_{2}$ for every other excitation. Images are acquired within a coronal plane without slice selection.

A HyperCEST CSI sequence according to the sequence diagram shown in Fig. 3 was implemented and applied in 3 of the 10 rats after CB6 application. A saturation train of 10 rectangular 50 ms long saturation pulses (∼24 μT) separated by 1 ms long gaps in each TR interval was used. Saturation frequency was alternatingly shifted to 122/282 ppm. Phase-encoding started in k-space center after 3 initial dummy acquisitions. The same excitation pulse as for z spectroscopy was used. Sequence parameters: TR 3 s (minimum, respiratory trigger), flip angle 90°, bandwidth 30 kHz, 1024 points, FOV 56 mm × 84 mm, acquired matrix 8 × 12, reconstructed matrix 16 × 24, no slice selection.

### 2.4 Data analysis

Reconstruction and processing of ^129^Xe MR data was performed in GNU Octave (version 6.4.0, The Octave Project Developers).

#### 2.4.1 HyperCEST z spectroscopy

Data from z spectroscopy were apodized using a squared von Hann window function and Fourier transformed. Spectra were phase-corrected to zeroth order and the real parts of the spectra integrated to quantify ^129^Xe in lipids (190.2 to 193.6 ppm), aqueous tissues (195.8 to 203.9 ppm, TP) and red blood cells (208.6 to 215.9 ppm, RBC). Data were rescaled assuming monoexponential $T_{1}$ decay with $T_{1}$ within the ^129^Xe reservoir determined by the median ratio of observed TP signals outside the central region of direct excitation within both averages. Obtained z spectra were normalized by the average of the 3 data points with saturation at largest chemical shift.

A Lorentzian depolarization function of the form (adapted from [26], [29], neglecting baseline depolarization)

(1) $$f(\delta )=\exp\left(-t_{\mathrm{sat}}\frac{\lambda \frac{\Gamma^{2}}{4}}{\frac{\Gamma^{2}}{4}+{\left(\delta -\delta_{0}\right)}^{2}}\right)$$

was fitted to the z spectra obtained before CB6 application in the range 118 ppm to 285 ppm in order to determine the mean center frequency $\delta_{0}$ of the resonances in z spectra associated with direct excitation. The parameter $t_{\mathrm{sat}}$ here denotes the saturation time of 1 s, λ the on-resonance depolarization rate and Γ the full-width at half-maximum.

HyperCEST asymmetry (HCA) was computed analogously to MTRasymm [30] from interpolated z spectra as

(2) $$\mathrm{HCA}(\delta_{0}-\Delta \delta )=m(\delta_{0}+\Delta \delta )-m(\delta_{0}-\Delta \delta )$$

with *m* the normalized ^129^Xe signal, $\delta_{0}$ the center frequency of the resonances in z spectra before CB6 averaged over the group and Δδ the chemical shift difference between saturation pulse and center frequency of the resonance. HCA was smoothed using a 20 ppm boxcar window. HCA was evaluated at 120 ppm for statistical comparisons, which is the frequency where the center of the reduction of signal due to saturation of ^129^Xe in CB6 is expected [24], [25]. This corresponds to a chemical shift difference approximately 80 ppm between the dissolved-phase resonances and CB6.

#### 2.4.2 HyperCEST CSI

Voxelwise fits of a function *f* consisting of a sum of three complex Lorentzians to CSI spectra were performed to investigate local differences of the HyperCEST effect in the individual compartments,

(3) $$f(\delta )=\overset{3}{\underset{j=1}{\sum }}\frac{1}{\pi }\left(\frac{\gamma_{j}/2}{{\left(\gamma_{j}/2\right)}^{2}+{\left(\delta -\delta_{0,j}\right)}^{2}}-i\frac{\delta -\delta_{0,j}}{{\left(\gamma_{j}/2\right)}^{2}+{\left(\delta -\delta_{0,j}\right)}^{2}}\right)M_{j}e^{i\phi_{j}}\text{.}$$

In order to improve the convergence of the fitting routine, fits were performed iteratively within averaged and progressively smaller voxels and results used as starting values in the next iteration. This algorithm started with one averaged voxel for the whole image and terminated when the reconstructed voxel size was reached.

The complex scale factors $M_{j}e^{i\phi_{j}}$ from the fitted Lorentzians were attributed to the respective compartments based on the frequency range of the determined center frequency $\delta_{0,j}$. The products $M_{j}e^{i\phi_{j}}$ were summed if the fit converged to a function with multiple Lorentzians within the same frequency range. Attenuation for each compartment was then computed from the resulting intensity *I* in a voxel as

(4) $$1-\frac{I(\Delta \delta =-80\;\mathrm{ppm})}{I(\Delta \delta =+80\;\mathrm{ppm})}$$

in order to form corresponding attenuation maps with Δδ the chemical shift difference between saturation pulse and center frequency of the resonance.

### 2.5 Statistical analysis

Statistical analysis was performed using R (The R Foundation for statistical computing, version 4.1.2). Wilcoxon signed-rank tests were performed for comparison of HCA at 120 ppm before CB6 administration against zero, before and after CB6 administration as well as to compare HCA at 120 ppm in individual chemical compartments. The significance level was set to 0.05 two-sided.

## 3 Results

**Figure 4:**
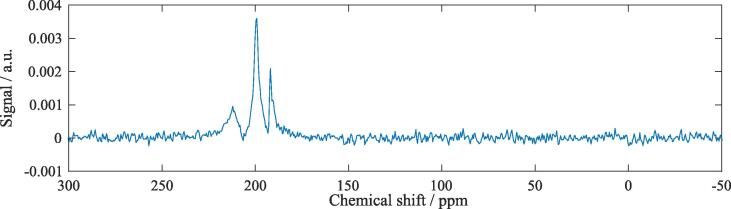
Exemplary MR spectrum (real part) with saturation frequency at 373 ppm before CB6 application in rat 8. Three dissolved-phase resonances associated with the compartments RBC, TP and lipids (decreasing chemical shift) are visible whereas no clear ^129^Xe signal from the gas phase at ∼0 ppm is discernible in this example, likely as a consequence of frequency-selective excitation. SNR determined by the ratio of peak height and standard deviation of the noise is estimated as 12 for RBC, 46 for TP and 27 for lipids in this example.

Fig. 4 shows a representative MR spectrum before CB6 application. At least three resonances in the ^129^Xe dissolved phase around 200 ppm are clearly visible associated with ^129^Xe in the three phases RBC, TP and lipids whereas gaseous ^129^Xe is not discernible.

**Figure 5:**
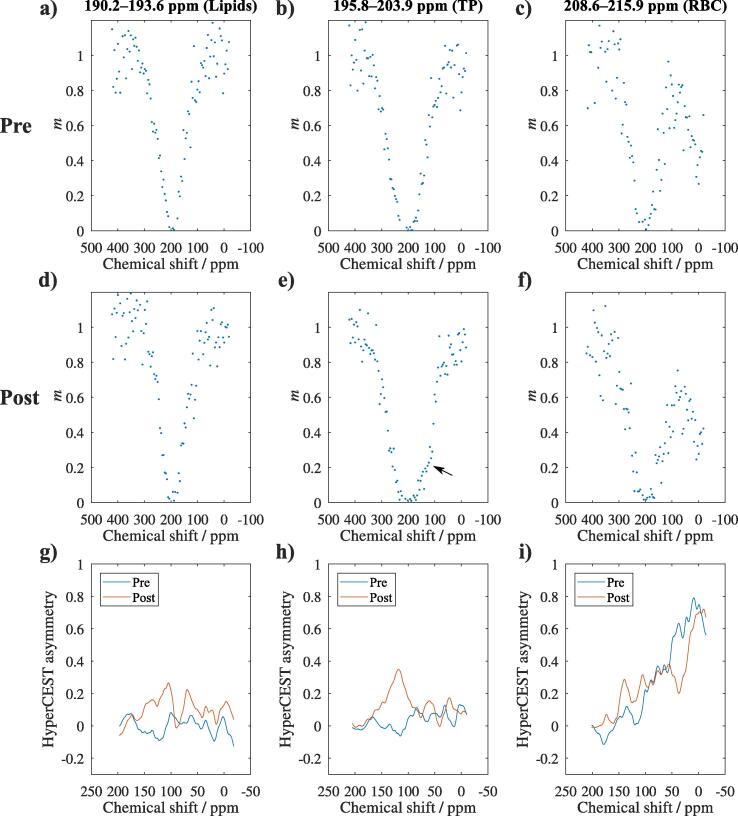
Exemplary z spectra and HCA curves in rat 8. a) to c) show z spectra before application of CB6 (dots) integrated in the spectral ranges corresponding to lipids (190.2–193.6 ppm), TP (195.8–203.9 ppm) and RBC (208.6–215.9 ppm). d) to f) similarly show data after CB6 application. A reduction of ^129^Xe signal becomes apparent in the range of 120 ppm mainly in the TP data (arrow). Noise appears to increase at frequencies further away from 200 ppm, which may be partly due to the rescaling of ^129^Xe signals for compensation of $T_{1}$ decay. HCA depicted in g) to i) is increased post CB6 at 120 ppm, with the most prominent peak again in the TP data.

HyperCEST z spectra before and after application of CB6 in the same animal are shown in Fig. 5. The appearance is largely symmetric before CB6 application for lipids and TP whereas a clear second resonance is visible at ∼0 ppm associated with gaseous ^129^Xe in the case of RBC. Mainly the z spectra of the TP resonance show a clear reduction of ^129^Xe signal at the CB6 frequency of ∼120 ppm.

Average center frequencies $\delta_{0}$ of the model functions fitted to the z spectra before application of CB6 were 196.8 ppm for the lipid resonance, 204.9 ppm for the TP resonance and 201.0 ppm for the RBC resonance. Average fitting results for full-width at half-maximum Γ were 50 ppm, 51 ppm, and 57 ppm for lipids, TP and RBC, respectively. HCA curves calculated using the mentioned mean center frequency of the respective resonance are also shown in Fig. 5.

**Figure 6:**
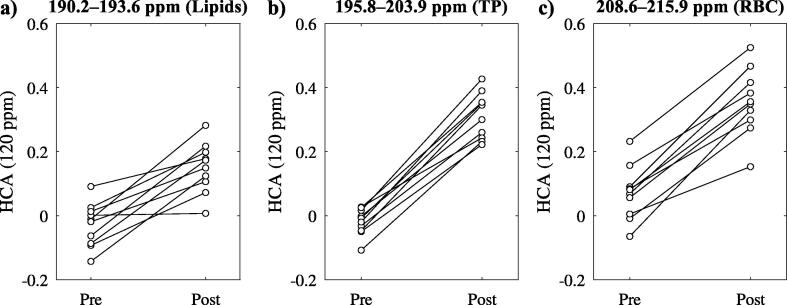
Comparison of HCA from z spectroscopy at 120 ppm before and after CB6 application. HCA at 120 ppm was significantly different from zero already before CB6 application in the case of red blood cells (RBC), p=0.027, but not for lipids and aqueous tissues (TP). Significant changes are observed for all resonances, p=0.002 in each case.

Fig. 6 shows data of HCA at 120 ppm before and after CB6 application. HCA was not significantly different from zero at baseline for lipids and TP but for RBC, p=0.027. HCA increased significantly after CB6, p=0.002 for all resonances. HCA post CB6 application was lower in lipids compared to TP, p=0.002, and RBC, p=0.004.

**Figure 7:**
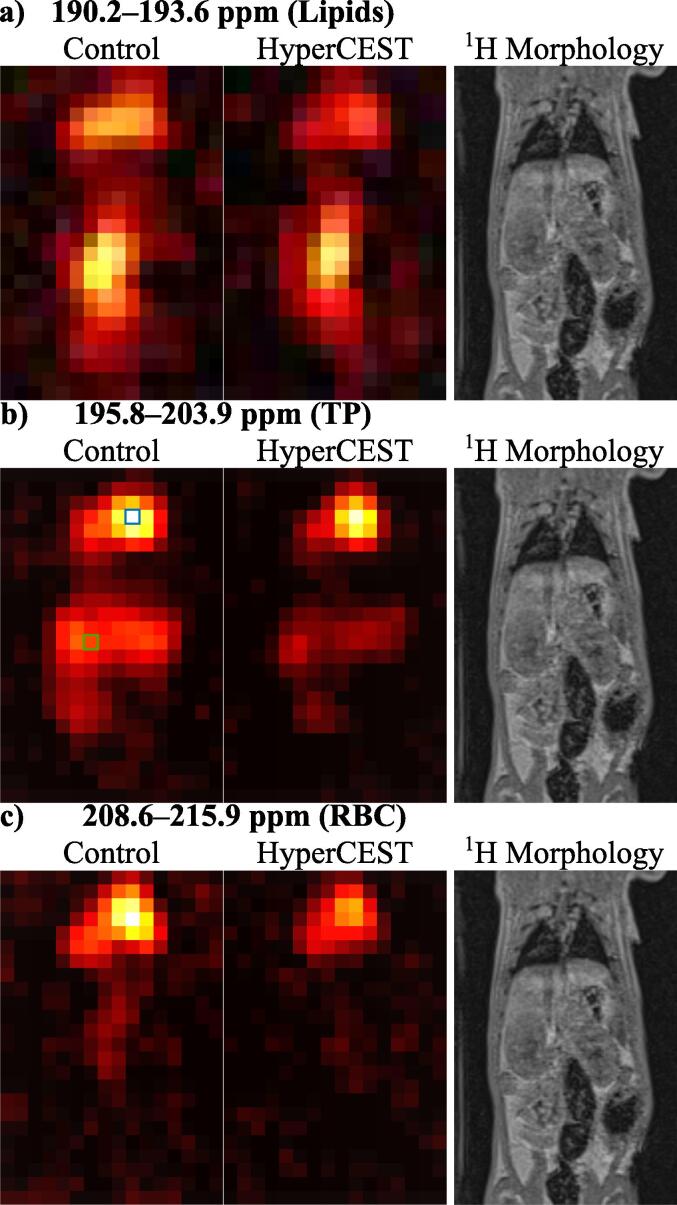
Spectral maximum intensity projections of magnitude CSI data along with a slice from ^1^H morphological acquisition in rat 8 between a) approximately 190.2 ppm and 193.6 ppm, b) 195.8 ppm and 203.9 ppm and c) 208.6 ppm and 215.9 ppm, corresponding roughly to ^129^Xe in lipids, aqueous tissues (TP) and red blood cells (RBC). Broad resonances, e.g., in lung tissue may lead to some spectral leakage. Magnitude spectra from voxels marked by blue and green box (arbitrarily shown in the TP data) are displayed in Fig. 8a)/b), respectively. RBC signal is visible in the aorta in the control but not in the HyperCEST acquisition.

**Figure 8:**
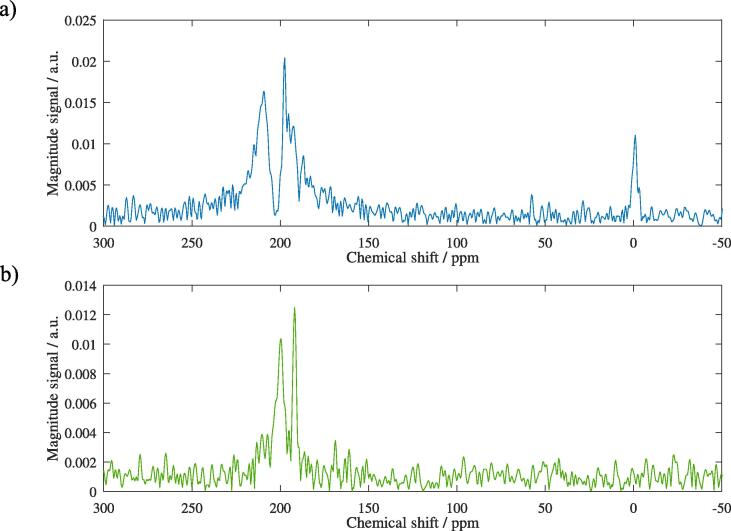
Magnitude spectra from control HyperCEST CSI acquisitions in voxels placed within the thorax (a), blue) and within the abdomen (b), green). Broad resonances within the dissolved phase in the region around 200 ppm are visible in the thorax voxel (a) with an additional resonance at ∼0 ppm associated with gaseous ^129^Xe. The spectrum in b) shows a sharp resonance at ∼190 ppm associated with lipid-dissolved ^129^Xe.

Spectral maximum intensity projections from HyperCEST CSI acquisitions as well as morphologic ^1^H MR images for comparing the anatomy are shown in Fig. 7. Clearly discernible organs and structures within the CSI images besides the lungs are the heart and kidneys, as well as the aorta and mesenteric fat. Most pronounced signal reduction is apparent in the spectral range above 195 ppm whereas the change in the spectral region of 190.2–193.6 ppm is mostly confined to the thorax. Fig. 8 shows MR spectra of CSI in the voxels outlined in Fig. 7.

**Figure 9:**
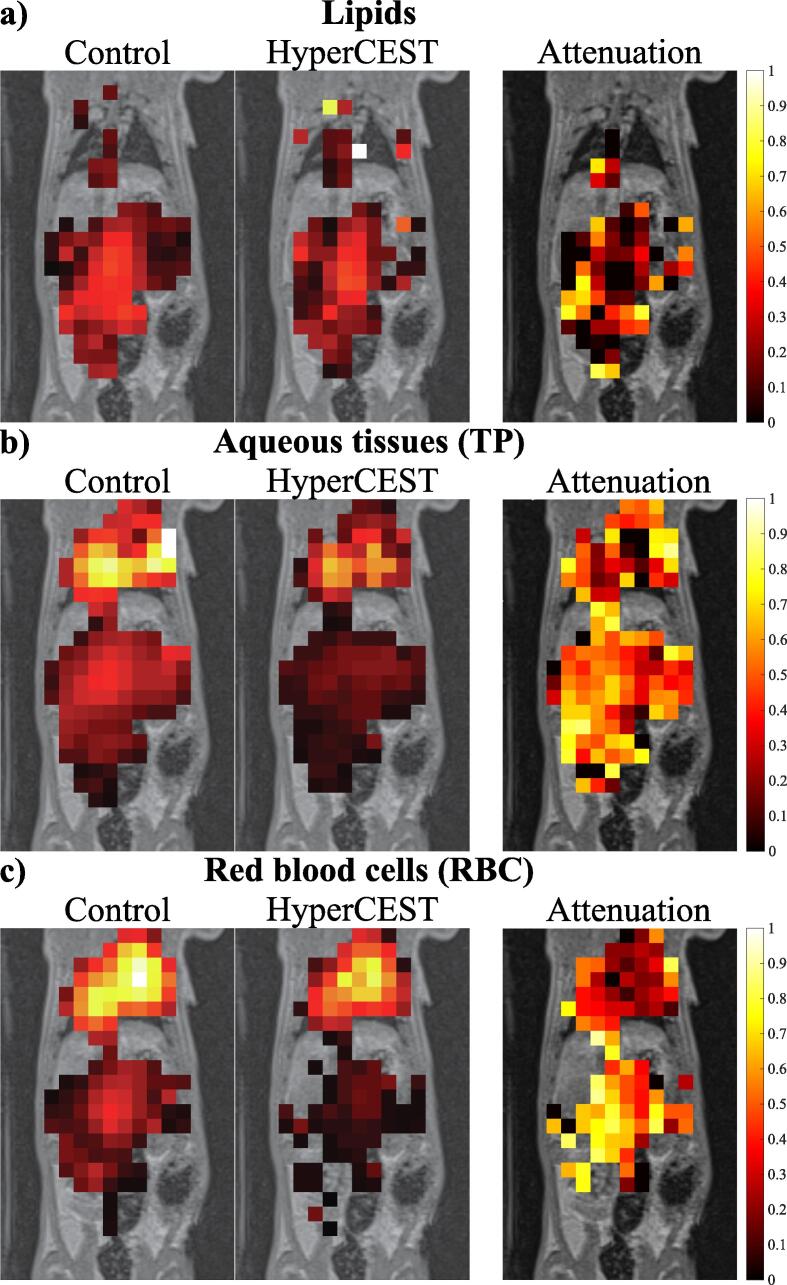
Summed scale factors of fitted Lorentzians corresponding to ^129^Xe in a) lipids, b) TP and c) RBCs with attenuation maps overlaid over a slice from ^1^H morphological acquisition. Transparent voxels in ^129^Xe MRI do not contain signal in the respective resonance as judged by the frequencies of the fitted Lorentzians. Transparent voxels in attenuation maps indicate that at least one voxel in the intensity maps does not contain signal. The maps suggest differing attenuation between compartments as well as a gradient with lower ratios near the lung region, particularly in the case of the RBC resonance, potentially caused by inflow of fresh ^129^Xe during the saturation train.

Fig. 9 shows results from Lorentzian fits in CSI acquisitions. The fitting results suggest the presence of a gradient in HyperCEST effect intensity along the vertical image axis in the case of RBC and possibly TP phase whereas for lipids, there seems generally almost no effect.

## 4 Discussion

Purpose of this study was to investigate compartmental differences of HyperCEST effects of the previously established CB6 HyperCEST contrast agent, to develop strategies to mitigate the influence of ^129^Xe signal intensity drifts on HyperCEST, and to investigate the feasibility of HyperCEST z spectroscopy and CSI in spontaneously breathing rats. Further, among the strategies to mitigate signal instability were centric reordering of saturation frequencies around the ^129^Xe dissolved-phase resonances as shown in Fig. 2b, the introduction of the quantity HCA for data analysis and long-term signal averaging.

We found the presence of large differences in the effect on the individual chemical compartments, in particular the very small effect on ^129^Xe in lipids. This may be due to the very high affinity of ^129^Xe to lipids and consequently comparatively low fraction of ^129^Xe within the CB6 cavity at a given instant of time although other effects like CB6 solubility may also play a role. Since the CB6 molecule has both a hydrophobic interior essential for ^129^Xe binding and hydrophilic rims [31], the exact mechanisms involved seem somewhat unclear. As a consequence of these large differences, proper knowledge of the chemical compartment probed as provided by CSI sequences seems vital in the interpretation of HyperCEST MRI in vivo.

Another important result of our study is the establishment of the feasibility of ^129^Xe HyperCEST z spectroscopy in the presence of signal intensity drifts associated with spontaneous respiration. The proposed quantity HCA shows significant changes after CB6 application and seems robust against signal drifts occuring during extended measurements, e.g., due to changes of respiration rate and tidal volume unavoidable under spontaneous respiration as well as changes of heart rate or potentially time-varying $T_{1}$ relaxation due to variable oxygen concentration in the lungs and blood oxygenation on which the ^129^Xe $T_{1}$ in blood strongly depends [32].

The fact that HCA at 120 ppm is different from zero at baseline in the case of the RBC resonance is likely caused by interaction with gaseous ^129^Xe, similar to previous results from thoracic imaging experiments employing saturation of slabs of gaseous ^129^Xe in the lung [33]. Consequently, the assumption of symmetry in the analysis of ^129^Xe z spectra may not always be valid in vivo. Nonetheless, at least for TP and lipids and using the proposed saturation train, the additional resonance in the region of the gas phase near ∼0 ppm in the z spectra seems to be weak and confined to a region of chemical shift outside the range of saturation frequencies of interest for the detection of CB6. Consequently, measurements after CB6 administration alone could be sufficient to assess the HyperCEST effect of CB6. The influence of the interaction with gaseous ^129^Xe should also be much lower in regions outside the thorax since during the saturation train length in our HyperCEST CSI sequence ^129^Xe atoms initially in the gas phase are unlikely to leave the thorax.

Imaging shows most pronounced HyperCEST effects of CB6 in the abdomen and aorta for TP and RBC. Exchange with gaseous ^129^Xe seems to create a gradient with increasing attenuation further away from the lungs, meaning the HyperCEST effect could be more challenging to detect in certain anatomical regions, depending on the route of ^129^Xe administration.

The HyperCEST effect in lipids in spatially non-selective z spectroscopy may in fact even be overestimated due to leakage from broad resonances in the lung as well as exchange with the other compartments during the saturation train. The relatively strong signal and apparent reduction of signal in the range 190.2–193.6 ppm associated with lipids in the thorax region in CSI is likely mostly due to spectral leakage from the broad TP resonance as a consequence of the numerous magnetic susceptibility jumps in lung tissue.

Although the failure to create a noticable HyperCEST effect in fatty tissues and certain anatomical regions may seem disappointing in the light of the vision of functionalized HyperCEST MRI as a clinical molecular imaging method, this is likely to depend on the specific host structure used. It also means at the same time that there is a new form of contrast generation, which, for example, increases the relative signal contribution of fatty tissues. This may be desirable, e.g., when performing ^129^Xe thermometry in lipids which may be of interest for various reasons, among them the very strong temperature dependence of the ^129^Xe chemical shift [28]. Other methods to selectively image ^129^Xe in fat like the use of high echo times or spin echoes are feasible but associated with detrimental effects like increased scan time or the destruction of hyperpolarization.

One could also envision potential applications to lung imaging like simultaneous ventilation/perfusion assessment using methods similar to dynamic contrast-enhanced ^1^H MRI by detecting the HyperCEST effect in the TP or gaseous ^129^Xe phase. This could have advantages over conventional dynamic–contrast enhanced MRI which is not specific for the lung capillary bed, i.e., gas-exchange region as opposed to nuclear-medicine methods clinically well established for lung perfusion assessment [34]. In analogy to established procedures in ^1^H MRI, this would likely require rapid measurements and bolus injection of CB6, the latter being difficult in small animals. The strong ^129^Xe signals within the lung could facilitate such rapid measurements. A downside of the use of non-functionalized CB6 as contrast agent could be a reduction of $T_{2}$ in compartments which are in rapid exchange with CB6 although we observed no severe line broadening in our z-spectroscopy data after CB6 application.

The concentration of ∼5 mM of CB6 used in this study is fairly high and the requirement of high concentrations is certainly detrimental for potential future molecular imaging applications employing functionalized molecules which may not be obtainable or toxic in such amounts. Potentially also smaller concentrations could be detected at higher $B_{1}$ amplitudes, which however—depending on the exchange conditions in the various chemical compartments—could lead to an increased broadening in the z spectra, an effect limiting the useful range of $B_{1}$ amplitudes [26]. A further, general difficulty for translation of the presented methods to larger animals or humans could also be that sufficient $B_{1}$ amplitudes and $B_{1}$ integrals may in practice not be possible to obtain with currently available RF instrumentation for human-scale ^129^Xe MRI or measurements could otherwise be limited by constraints on specific absorption rates. However, there is likely still potential for optimization of the saturation train with respect to minimization of specific absorption rate and direct saturation while maintaining saturation efficiency. For this, future work should investigate the exchange rates in vivo which likely deviate from previous in vitro values due to competing guests.

### 4.1 Study limitations

One of the limitations of this study is that the concentration of CB6 in the injected solution is not accurately known. Due to the limited solubility of CB6, undissolved CB6 was removed by filtration. The alternative would have been to inject an apparently oversaturated solution as in previous work [25], which may be associated with the potential danger of embolism. The effect of such undissolved particles on the imaging results remains uncertain.

Due to limited sample size, we could not show the sensitivity of the proposed HyperCEST CSI sequence to the presence of CB6 on statistical grounds. However, as the way of contrast generation in the z spectroscopy sequence is essentially identical to the HyperCEST CSI sequence, the conclusions drawn should also apply to HyperCEST CSI.

Inaccuracies in transmitter calibration may lead to unintended effects since deviation of the flip angle of the nominal 90° excitation pulse could lead to a dependence of measurements on previous RF history. There is, however, no generally accepted method for ^129^Xe MRI transmitter calibration outside of the lung and in small animals, in particular. Additional saturation pulses after data acquisition destroying the whole ^129^Xe magnetization within the coil volume could potentially mitigate such issues.

A limitation of our data analysis is the simplistic assumption of mono-exponential $T_{1}$ decay within the ^129^Xe reservoir although it is known that the change of the surface-volume ratio of a deflating Tedlar bag in principle leads to more complicated decay [35], depending on flow rate and several other parameters.

Finally, the determination of the amounts of ^129^Xe within the individual compartments in z spectroscopy through numerical integration may be affected by spectral leakage as it also becomes evident in the analysis of the CSI data. Fitting a sum of complex Lorentzians to the whole-body spectra proved to be difficult, likely due to magnetic field inhomogeneities and associated non-Lorentzian lineshapes. Field inhomogeneities present within the FOV may also influence the size of the HyperCEST effect in CSI data as only two discrete saturation frequencies were used. We expect field inhomogeneities to be relatively small compared to the width of the resonance associated with CB6 apparent in z spectra, however.

## 5 Conclusions

HyperCEST using CB6 as a tracer molecule may produce very different contrasts in different chemical compartments and anatomical regions in vivo. Our study shows the feasibility of HyperCEST z spectroscopy and imaging in spontaneously breathing rats.

Provided suitable functionalized molecules can be found, clinical HyperCEST imaging should be feasible in specific regions of the body. Although the clinical value of HyperCEST using non-functionalized molecules is not clear, it may enable suppression of unwanted signals or facilitate contrast agent dynamics not achievable with hyperpolarized ^129^Xe alone.

## Declaration of Competing Interest

The authors declare that they have no known competing financial interests or personal relationships that could have appeared to influence the work reported in this paper.
